# Supplementary material for: CRISPR spacers acquired from plasmids primarily target backbone genes, making them valuable for predicting potential hosts and host range
Source: Microbiol Spectr. 2024 Nov 7;12(12):e00104-24. doi: 10.1128/spectrum.00104-24 (PMC11619364; doi:10.1128/spectrum.00104-24)

**SUPPLEMENTARY FIGURES**

**Fig. S1.** Accuracy of CRISPR-based method at each taxonomic level when using threshold of 90% ID. For each taxonomic level, the percentage of unique plasmids (solid columns) and of all the plasmids in the PLSDB (dotted columns) for which the prediction of our method matched the known host in the database is shown.


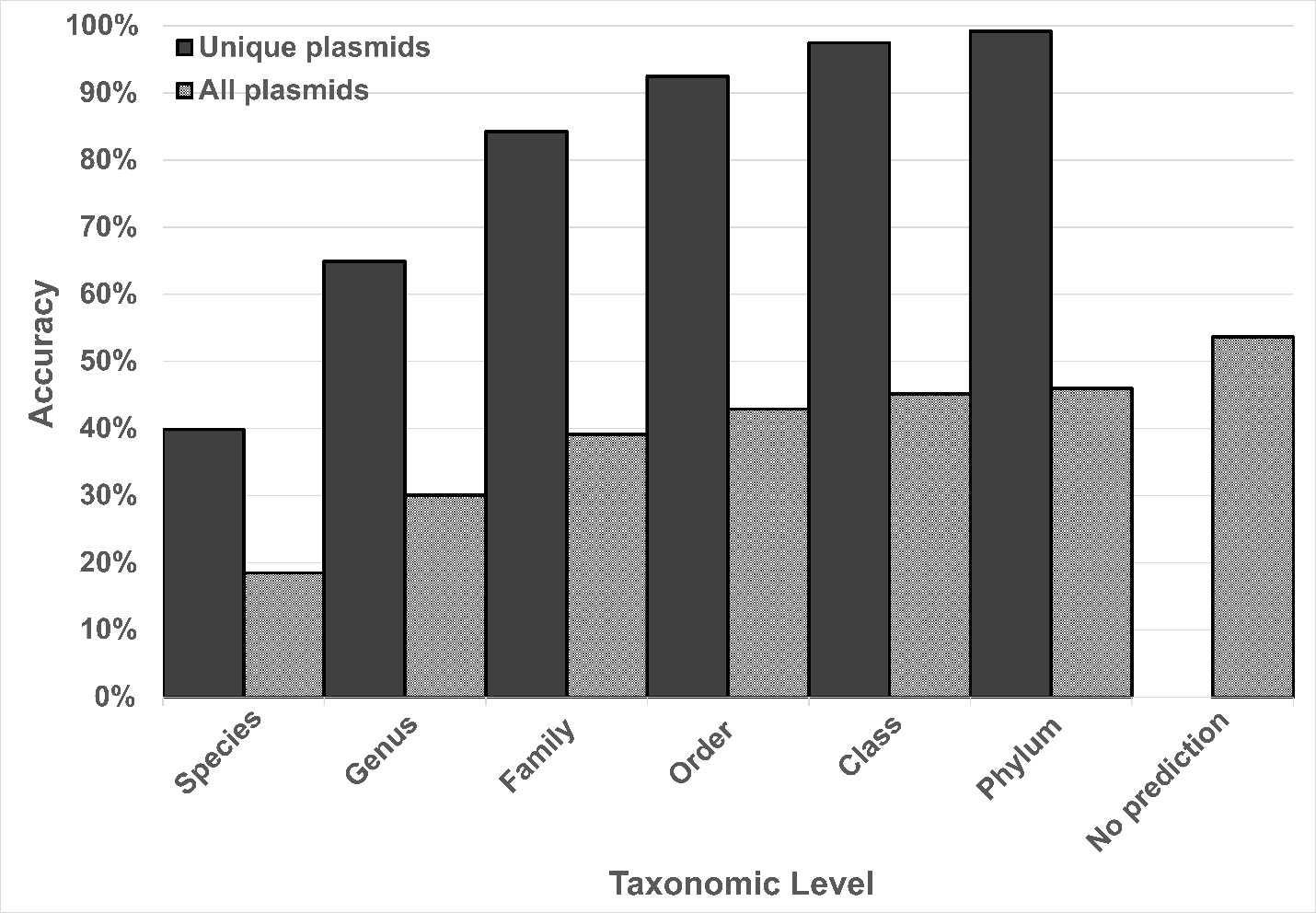


**Fig. S2.** Host range grades for mobile and non-mobile plasmids when using 90% ID. a) Percentage of each host range grade in all the matched plasmids (all, left bar), in mobile plasmids (MOB+, middle bar) and in non-mobile plasmids (MOB-, right bar). b) Percentage of mobile (MOB+, orange) and non-mobile (MOB-, gray) plasmids in each host range grade.


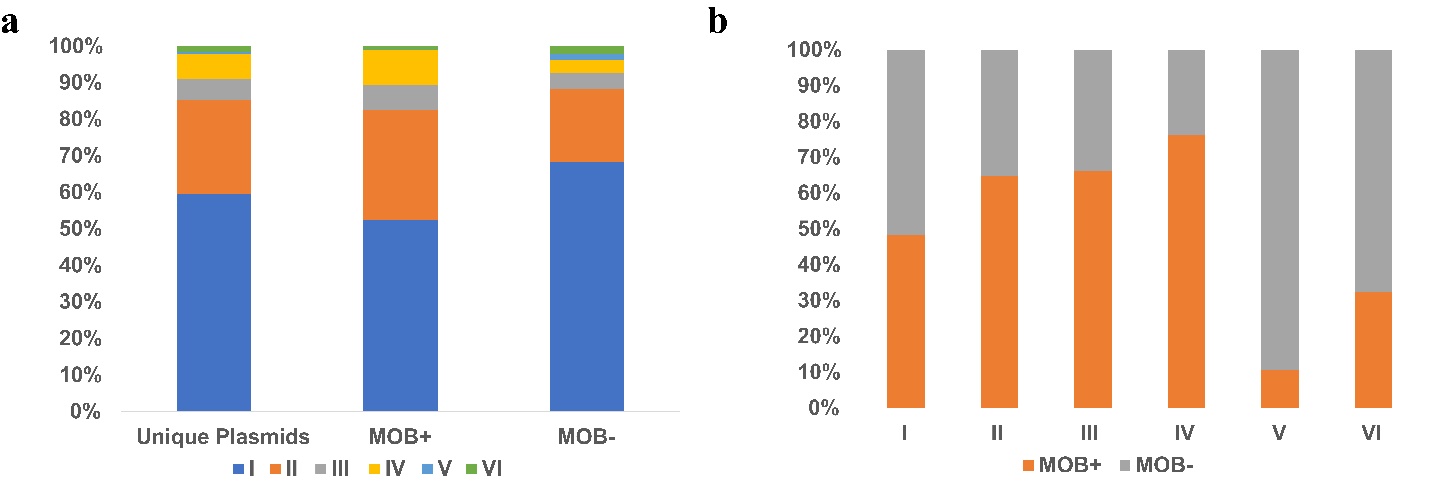

Supplement: Supplemental figures — Fig. S1 and S2. [file spectrum.00104-24-s0001.docx]
